# Impact of coaching on physician wellness: A systematic review

**DOI:** 10.1371/journal.pone.0281406

**Published:** 2023-02-07

**Authors:** Sylvain Boet, Cole Etherington, Pierre-Marc Dion, Chloé Desjardins, Manvinder Kaur, Valentina Ly, Manon Denis-LeBlanc, Cecile Andreas, Abi Sriharan

**Affiliations:** 1 Department of Anesthesiology and Pain Medicine, Hyperbaric Medicine Unit, The Ottawa Hospital, Ottawa, Canada; 2 Department of Innovation in Medical Education, The Ottawa Hospital, Ottawa, Canada; 3 Clinical Epidemiology Program, The Ottawa Hospital Research Institute, Ottawa, Canada; 4 Institut du Savoir Montfort, Ottawa, Canada; 5 Faculty of Education, University of Ottawa, Ottawa, Canada; 6 Francophone Affairs, Faculty of Medicine, University of Ottawa, Ottawa, Canada; 7 Health Sciences Library, University of Ottawa, Ottawa, Canada; 8 Professional and Continuing Studies, Royal Roads University, Victoria, Canada; 9 Institute of Health Policy, Management and Evaluation, University of Toronto, Toronto, Canada; National University of Singapore, SINGAPORE

## Abstract

Physician wellness is critical for patient safety and quality of care. Coaching has been successfully and widely applied across many industries to enhance well-being but has only recently been considered for physicians. This review aimed to summarize the existing evidence on the effect of coaching by trained coaches on physician well-being, distress and burnout. MEDLINE, Embase, ERIC, PsycINFO and Web of Science were searched without language restrictions to December 21, 2022. Studies of any design were included if they involved physicians of any specialty undergoing coaching by trained coaches and assessed at least one measure along the wellness continuum. Pairs of independent reviewers determined reference eligibility. Risk of bias was assessed using the Cochrane Risk of Bias Tools for Randomized Controlled Trials (RCTs) and for Non-randomized Studies of Interventions (ROBINS-I). Meta-analysis was not possible due to heterogeneity in study design and outcome measures as well as inconsistent reporting. The search retrieved 2531 references, of which 14 were included (5 RCTs, 2 non-randomized controlled studies, 4 before-and-after studies, 2 mixed-methods studies, 1 qualitative study). There were 1099 participants across all included studies. Risk of bias was moderate or serious for non-RCTs, while the 5 RCTs were of lower risk. All quantitative studies reported effectiveness of coaching for at least one outcome assessed. The included qualitative study reported a perceived positive impact of coaching by participants. Evidence from available RCTs suggests coaching for physicians can improve well-being and reduce distress/burnout. Non-randomized interventional studies have similar findings but face many limitations. Consistent reporting and standardized outcome measures are needed.

## Introduction

Physician burnout represents a profound and longstanding epidemic in healthcare, with rates reported to be 1.5 times higher than the general US working population (37.9% vs. 27.8%) [1]. The prevalence of physician burnout has only been exacerbated by the COVID-19 pandemic. According to a national study conducted in 2021 in Canada, 53% of physicians and medical learners experienced symptoms of burnout, nearly double what was reported in a 2017 study (30%) [2].

The rise in physician burnout has serious consequences for both patient safety and quality of care. According to a recent systematic review and meta-analysis, physicians with burnout are twice as likely to be involved in patient safety incidents, to exhibit low professionalism and to receive low satisfaction ratings from patients [3]. Burnout is also costly to the healthcare system, with the cost to replace one physician estimated at $500 000, taking into account hiring and training costs as well as productivity losses [4].

Although many different interventions have been implemented to address physician burnout, several systematic reviews have found a wide range in effectiveness, from modest improvement in, to worsening of, burnout [5–12]. Existing interventions have largely focused on a specific stressor (e.g., modifications to electronic health record documentation to reduce associated burnout [6, 7]) and are short in duration (e.g., self-care workshops, stress management skills training [9]), or unsustainable given the resource limitations of the healthcare system (e.g., reducing physician workload [9]). Interventions involving single or short-term training sessions do not show consistent evidence of effectiveness, and in several instances, have even worsened resilience upon completion [5]. Other interventions have limited capacity to generalize to all aspects of physicians’ lives. For example, wellness interventions for neurosurgical faculty and residents involved free gym memberships, group gym visits, and team-based exercise sessions [8]. Evidence also suggests that interventions perceived as adding “burden” to physicians’ lives (i.e., requiring a substantial time commitment outside of working hours) are unlikely to be effective [13]. These limitations highlight the need for interventions that are tailored to the complex and unique lived experiences of individual physicians, rather than imposing a top-down, one-size-fits-all approach. The most effective interventions are those designed to meet the needs of the individuals they serve [14, 15]. Furthermore, evidence suggests that physicians are typically reluctant or ashamed to access mental health services or perceive that their problems are “not severe enough” [16]. Thus, initiatives framed as proactive professional development (rather than reactive) may ultimately be more accessible and effective in preventing distress and burnout, rather than offering resources too late in the game.

Coaching has been found to improve well-being and reduce burnout across many industries (e.g., finance, nuclear power, education), and more recently, healthcare [17–19]. The International Coaching Federation (ICF) defines coaching as “a thought-provoking and creative process that inspires [participants] to maximize their personal and professional potential.” [20] Delivered by trained coaches, coaching is a proactive and action-oriented intervention intended to empower individuals to with self-discovery, strength-building and self-efficacy [20, 21]. Through powerful questions and other techniques such as embodiment, active listening, curiosity, or emotional literacy, trained coaches help individuals to act according to their core values, regain control over their lives and achieve fulfillment [20, 21]. Although coaching is emerging as a promising intervention for physician wellness, a comprehensive summary and appraisal of the available evidence has yet to be completed. This is necessary to guide healthcare organizations considering implementation of coaching to support physician wellness.

### Objectives

We aimed to conduct a systematic review of the existing evidence on the effect of coaching by trained coaches on physician wellness.

## Methods

### Protocol

This systematic review was planned and conducted according to A Measurement Tool to Assess Systematic Reviews (AMSTAR-2) standards [22] and reported in adherence to the Preferred Reporting Items for Systematic Reviews and Meta-Analyses (PRISMA) checklist [23]. The review protocol was developed a priori and published on Open Science Framework [24].

### Eligibility criteria

Studies of any interventional design were included if they involved licensed physicians or post-graduate trainees of any specialty undergoing coaching by trained coaches, delivered individually or to groups. Studies had to assess at least one outcome related to wellness (e.g., quality of life, resilience, psychological well-being), distress, and burnout [25] to be included. To be included, the coaching intervention had to meet the internationally recognized definition and core competencies described by the ICF (Table 1) [20, 21]. The ICF defines coaching as “a though-provoking and creative process that inspires [participants] to maximize their personal and professional potential” [20]. This was important to distinguish coaching from other interventions with which it is often confused (e.g., feedback, teaching, mentoring, peer support) [26], and was verified with specific screening questions during the reference selection process. We specifically excluded coaching interventions that did not meet the ICF standard definition or that were implemented as part of a multifaceted intervention. Studies involving medical students were also excluded. Conference abstracts, letters, editorials, and commentaries were not eligible for inclusion.

**Table 1:** International coaching federation coaching competencies [20, 21].

Competency	Definition
A. Building the foundation	
1. Demonstrating ethical practice	Understanding and consistently applying coaching ethics and standards.
2. Embodying a coaching mindset	Developing and maintaining a mindset that is open, curious, flexible and client centered.
B. Co-creating the relationship	
3. Establishing and maintaining agreements	Partnering with the client to create clear agreements about the coaching relationship, process, plans and goals. Establishing agreements for the overall coaching engagement as well as those for each coaching session.
4. Cultivating trust and safety	Partnering with the client to create a safe, supportive environment that allows the client to share freely. Maintaining a relationship of mutual respect and trust.
5. Maintaining presence	Being fully conscious and present with the client, employing a style that is open, flexible, grounded and confident.
C. Communicating effectively	
6. Listening actively	Focusing on what the client is and is not saying to fully understand what is being communicated in the context of the client systems and to support client self-expression.
7. Evoking awareness	Facilitating client insight and learning by using tools and techniques such as powerful questioning, silence, metaphor or analogy.
D. Cultivating learning and growth	
8. Facilitating client growth	Partnering with the client to transform learning and insight into action. Promoting client autonomy in the coaching process.

### Search strategy and information sources

The search strategy was developed by an experienced information specialist (VL) in close collaboration with the research team (S1 Appendix) and reviewed by a second information specialist as per Peer Review of Electronic Search Strategies (PRESS) guidelines [27]. The databases MEDLINE, Embase, ERIC, and PsycINFO via Ovid and Web of Science were searched without date or language restrictions; however, only studies published in English or French were included in the final review. We planned to include an appendix of potentially relevant studies published in other languages; however, none were identified. The reference lists of included studies were also searched for potentially relevant studies that may not have been identified in the initial search. The final list of included studies was reviewed by coaching experts (SB, MDL, CA, AS) to confirm its relevance and completeness.

### Study selection

Identified studies were uploaded to Covidence, a web-based systematic review software (Melbourne, Australia). Duplicates were detected and removed automatically by Covidence. A screening tool was developed by the research team, piloted, and iteratively refined until acceptable inter-rater reliability was established (minimum Cohen’s kappa = 0.7). The tool was designed to facilitate a judgement of “include” or “exclude” based on the pre-specified inclusion criteria described above. Pairs of independent reviewers (CD, PMD, CE, MK) first assessed titles and abstracts for eligibility, followed by the full texts of articles of included studies and those deemed “unclear”. Screening for inclusion at each level was always conducted in duplicate, with disagreements resolved by consensus or involvement of a third reviewer as needed (SB).

### Data extraction

A data extraction form was developed and piloted, then used by the reviewers in duplicate to extract relevant information with Covidence. Extracted data included publication details (e.g., first author name, year of publication, country of data collection), study characteristics (e.g., study design, sample size, inclusion criteria), physician demographics, healthcare setting (e.g., community or academic hospital, specialty), intervention and comparator details, and the effect of intervention on reported outcomes of interest.

### Risk of bias

Pairs of independent reviewers (CD, PMD, CE, MK) assessed included studies for risk of bias using the Cochrane Risk of Bias Tool 2 (RoB2) [28], and for Non-randomized Studies of Interventions (ROBINS-I) [29], as appropriate. The RoB2 tool assesses the potential for bias in terms of trial design, conduct, and reporting. Within each domain, there is a series of questions which facilitate a judgement about the risk of bias. The judgement can be “low”, “high”, or can express “some concerns.” [28], The ROBINS-I tool includes seven domains of bias: bias due to confounding, bias in selection of participants into the study, bias in classification interventions, bias due to deviations from intended interventions, bias due to missing data, bias in measurement of outcomes, and bias in selection of the reported result [29]. Within and across each domain, a judgement is made based on specified criteria as to whether there is a “low”, “moderate”, “serious”, or “critical” risk of bias [29].

### Data synthesis

A narrative synthesis of results was conducted. We planned to conduct a meta-analysis if appropriate; however, this was not possible based on the heterogeneity in study design and outcome measures as well as inconsistent reporting.

## Results

### Study selection

**Fig 1:**
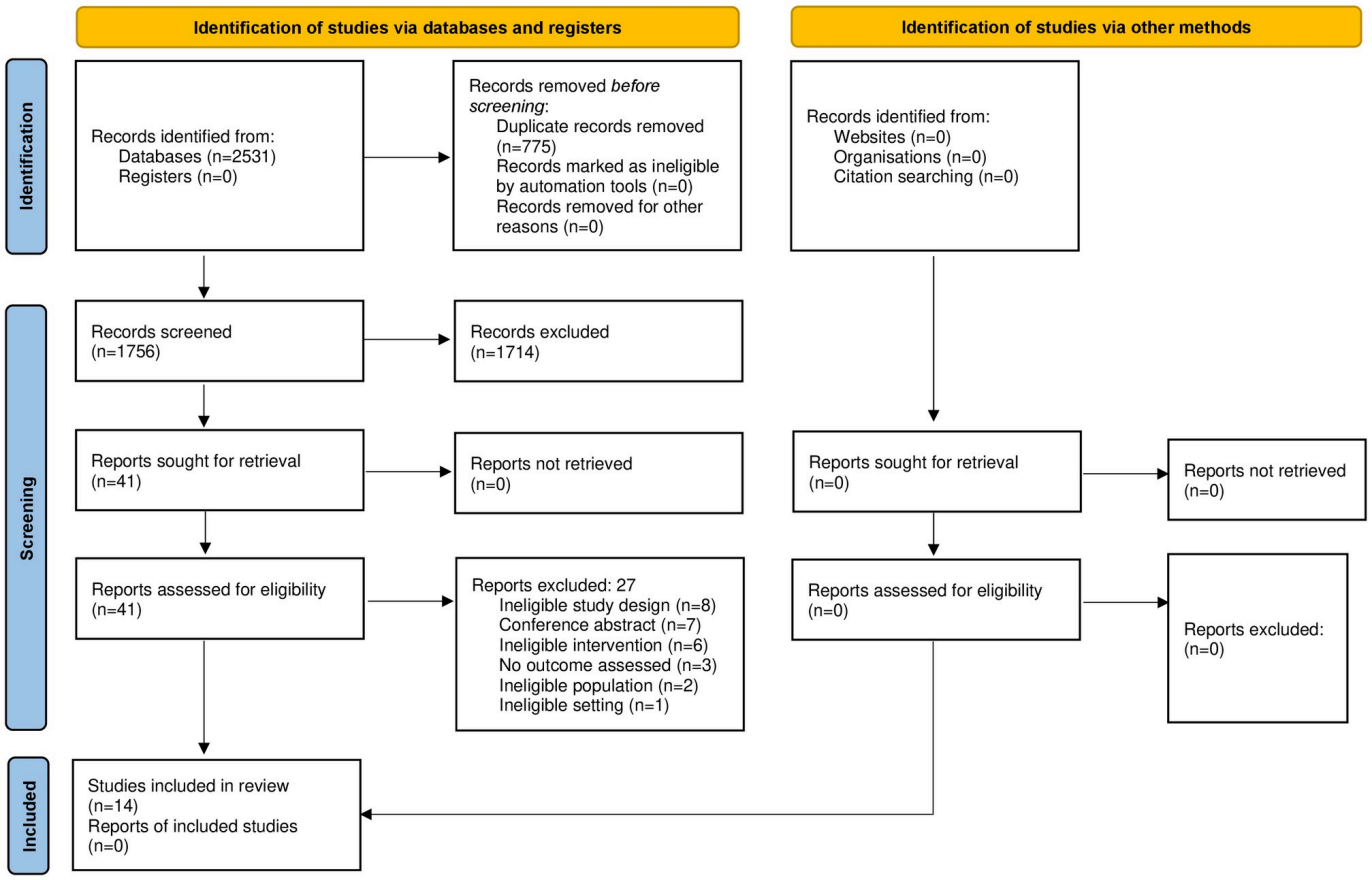
PRISMA flow diagram.

The literature search yielded 2531 studies. After removal of duplicates, 1756 studies were assessed for eligibility (Fig 1). Subsequently, 14 studies met the inclusion criteria and were included in this systematic review.

### Study and participant characteristics

Details on included study and participant characteristics are provided in Table 2. Of the 14 included studies, 5 (36%) were RCTs, 2 were non-randomized controlled studies, 4 (29%) were before- and after-studies, 2 (14%) were mixed-methods studies, and 1 (7%) was a qualitative study. There were 1099 participants across all included studies. Over half of the included studies (n = 8 [57%]) involved post-graduate trainees (n = 707 [64%]) as the recipients of the coaching intervention. Approximately half of participants were general practitioners or internal medicine specialists (n = 572 [52%]). Three studies did not report participant specialty (n = 158 [14%]). Of the 6 studies that reported participant age, the majority of participants were between 41 and 50. Across the 11 studies reporting participant sex, nearly two thirds of participants were male (n = 491 [57%]).

**Table 2:** Characteristics of included studies and participants.

First author, year, (country)	Study design	Sample size	Participants	Definition/description of coaching	Certification/training of coaches	Participant age (mean/median years) or Age groups (n/%)	Participant sex, No. (%)
de Lasson, [30] 2016, (Denmark)	Qualitative study	45	Staff physicians (speciality NR)	Coaching as a means to unfold a person’s potential in order to maximize performance.	“The medical consultant had a Master’s degree in Organizational Coaching. The nurses had academic degrees. One held a PhD in learning processes; the other a diploma in coaching from Cambridge University.”	NR	F: 38 (84.4) M: 7(15.6)
Dyrbye, [19] 2019 (United States)	Randomized controlled trial	88	Family medicine, general pediatrics, general internal medicine physicians	“Coaching is distinct from mentorship and peer support and involves inquiry, encouragement, and accountability to increase self-awareness, motivation, and the capacity to take effective action… Professional coaching can be tailored to focus on the aspects desired by recipients and can assist individuals in their effort to navigate their professional life, their choices, and the direction of their career.”	“Credentialed professional coaches were provided by Bluepoint Leadership Development Inc, an established international professional coaching company with experience coaching physicians.”	Intervention group: 31–40: 7 (15.9%) 41–50: 25 (56.8%) 51–60: 12 (27.3%) >60: 0 Control group: 31–40: 7 (16.7%) 41–50: 20 (47.6%) 51–60: 12 (28.6%) >60: 3 (7.1%)	F: 48 (54.5) M: 40 (45.4)
Dyrbye, [31] 2022 (United States)	Randomized controlled trial	80	Surgical specialties: cardiology, gastrointestinal, general, gynecologic, neurological, oncology, ophthalmologic, orthopedic, otorhinolaryngologic, pediatrics, plastic, trauma, urologic, vascular, thoracic, transplant, other (e.g. oral)	Professional coaching is "tailored to the individual’s needs and assists individuals in navigating professional choices and behaviors… Professional coaching allows individuals to identify opportunities, generate goals, and take action related to professional needs, such as influencing change, dealing with conflict, addressing detrimental aspects of work, improving career fit, and improving work-life integration."	“The coaches had received training from various organizations with robust offerings including Right Management Global Coaching Program, Columbia University Executive Coaching Program, the Association for Professional Executive Coaches and Supervisors, and the Institute of Leadership and Management Coaching Program resulting in a coaching certification by the training organization and/or the International Coach Federation. The coaches had 15 years to over 25 years of coaching experience and 7 to 15 years of experience specifically coaching physicians and surgeons.”	Intervention group: 31–40: 12 (30%) 41–50: 21 (52.5%) 51–60: 7 (17.5%) 61+: 0 Control group: 31–40: 18 (45%) 41–50: 12 (30.0%) 51–60: 9 (22.5%) 61+: 1 (2.5%)	F: 32 (40%) M: 48 (60%)
Fainstad, [32] 2022 (United States)	Randomized controlled trial	101	Residents across 12 programs (specialties NR)	“Professional coaching uses inquiry around perceptions, beliefs, and habits to define, reframe, and align work with personal values.”	“Coaches were certified by The Life Coach School, a thought-based coaching institution with training in both group and individual coaching.”	Intervention group: Mean (SD): 29.1 (2.3) Median (Range): 29 (25–35) Control group: Mean (SD): 29.6 (2.2) Median (Range): 29 (26–35)	F: 101 (100%)
Gardiner, [33] 2013, (Australia)	Non-randomized experimental study	69	General practitioners	“A cognitive behavioural coaching program advertised as a work-life balance retreat by the Rural Doctors Work-force Agency (RDWA).”	Two qualified coaching psychologists	NR	NR
Kakarala, [34] 2018 (United States)	Pre-post study design	12	Post-graduate trainees (specialty NR)	“The Massachusetts General Hospital (MGH) Professional Development Coaching Program (PDCP) was designed to optimize resident performance through principles of positive psychology, using a strength-based model.”	Certified Health and Wellness Coaches trained by Coaching Program Director. The training was 2 hours, and “combined theory and real-life practice utilizing positive psychology, mindful listening, self-reflection, exploration of possible solutions, and validation of the coachee’s self-identified strategies to reach goals.”	NR	F: 4 (33.3) M: 8 (66.7)
McGonagle, [35] 2020, (United States)	Randomized controlled trial	58	General practitioners	“Coaching is a one-on-one intervention between a coach and individual coachee that is systematic, collaborative, future-focused, and goal-focused, and is meant to help coachees attain valued professional or personal development outcome.”	Credentialing organizations included the Center for Credentialing and Education, the Center for Creative Leadership, International Coach Federation, Coaches Training Institute, and Wellcoaches.	Mean +/- Standard deviation: Intervention group: 43.31 +/- 8.76 Control group: 41.83+/-7.42	Intervention group: F: 21 (72.4) M: 8 (27.6) Control group: F: 25 (86.2) M: 5 (13.8)
McKimm, [36] 2018, United Kingdom	Mixed-methods study (pre/post design + qualitative interviews)	52	General practitioners	“The purpose of the coaching was to support [participants] in their decision-making processes and prepare them for any transition… Coaches worth with their clients to meet their need to reflect, practice, step back or forge ahead with changes they wanted to make.”	“Coaches belonged to a national network of experienced, executive, professional coaches who work within professional codes of ethics. The coaches were all trained professionals (2 with mental health backgrounds) well versed in managing the boundaries between coaching and therapy and could therefore direct [participants] towards counselling/therapy where relevant.”	Range: 30–50	F: 36 (69.2) M: 16 (30.8)
Palamara, [37] 2015 (United States)	Pre-post study design	72	Post-graduate trainees, internal medicine	“Coaching was based on the principles of positive psychology…Positive psychology coaching uses a strengths approach that emphasizes engagement, meaning, and accomplishment.”	“All coaches participated in 2 hours of training. Coaches were introduced to core concepts of developmental coaching and positive psychology, using hands on experience of coaching exercises. Strategies for managing particular situations such as poor intern performance and unrealistic self-assessment were reviewed.”	NR	NR
Palamara, [38] 2018 (United States)	Pre-post study design	104	Post-graduate trainees, internal medicine	“Positive psychology coaching, using a strengths-based approach, provides participants with additional tools required to cope with… personal and professional stressors.”	Coaches trained in the Professional Development Coaching Program using positive psychology and coaching principles.	NR	NR
Palamara, [39] 2021 (United States)	Pre-post study design	129	Post-graduate trainees, internal medicine	“Coaching focused on strengths evaluation, resiliency, and constructing a framework of positive well-being.”	“Coaches were provided a 3-hour skills-based training in positive psychology and coaching principles and given curricular guides for 1:1 meetings with their coachees.”	NR	F: 56 (43.8) M: 72 (56.2)
Palamara, [40] 2022 (United States)	Randomized controlled trial	150	Surgical residents (specialties NR)	Coaching uses “principles of positive psychology [and focuses on] creating an effect coaching presence, core coaching skills of listening, goal-setting, [and] asking future-oriented questions rather than giving advice.”	Coaches participated in a 3-hour training program from the Institute of Coaching and had the option to participate in two additional 90-minute refresher training sessions	Mean (SD): Intervention group: 30.3 (3) Control group: 30.7 (2.9)	F: 150 (100%)
Solms, [41] 2021 (Netherlands)	Non-randomized experimental study	114	Post-graduate trainees, pediatrics	“A result-oriented, systematic process in which the coach facilitates the enhancement of life experience and goal attainment in the personal and/or professional life of clients.”	Senior level of coaching experience, their experience with physician-clients, positive references from previous physician clients, and accredited coaching training.	Median (interquartile range): Intervention group: 33 (9.5) Control group: 35 (12)	Intervention group: F: 47 (82.5) M: 10 (17.5) Control group: F: 42 (73.7) M: 15 (26.3)
Song, [42] 2020 (United States)	Mixed-methods study (pre/post design + qualitative interviews)	25	Post-graduate trainees; General Surgery, Vascular Surgery, Cardiac Surgery, Plastic Surgery, Urology	Coaching focuses “on continued and evolving collaboration between coach and [participant]… The individual coaching sessions adapted to each [participant’s] needs at the time of the session.”	“The program was administered by a certified, professional coach.”	NR	F: 8 (32) M: 17 (68)

### Risk of bias

All five of the RCTs were found to be at low risk of bias across almost all domains (Table 3). The measurement of outcome domain and the missing outcome domain were found to be at some risk of bias for the Dyrbye et al. [19] and McGonagle et al. [35] RCTs, respectively. The Palamara et al. study [40] was found to have some concerns of bias for the deviations from intended interventions domain and the missing outcome domain. The included non-randomized interventional studies and quantitative portions of the mixed-methods studies were mostly at moderate or serious risk of bias across all domains (Table 4). The included qualitative study was not assessed for risk of bias.

**Table 3:** Risk of bias of included randomized controlled trials.

Author, year	Cochrane Risk of Bias 2 Assessment Domains					
	Randomization process	Deviations from intended interventions	Missing outcome data	Measurement of the outcome	Selection of the reported result	Overall bias
Dyrbye, [19] 2019	Low risk	Low risk	Low risk	Some concerns	Low risk	Some concerns
Dyrbye, [31] 2022	Low risk	Low risk	Low risk	Low risk	Low risk	Low risk
Fainstad, [32] 2022	Low risk	Low risk	Low risk	Low risk	Low risk	Low risk
McGonagle, [35] 2020	Low risk	Low risk	Some concerns	Low risk	Low risk	Some concerns
Palamara, [40] 2022	Low risk	Some concerns	Some concerns	Low risk	Low risk	Some concerns

**Table 4:** Risk of bias of included non-randomized interventional studies.

Author, year	ROBINS-I Assessment Domains							
	Confounding	Selection of patients into the study	Classification of interventions	Deviations form intended interventions	Missing data	Measurement of outcomes	Selection of the reported result	Total risk of bias
Gardiner, [33] 2013	Low risk	Low risk	Low risk	Low risk	Low risk	Low risk	Low risk	Low risk
Kakarala, [34] 2018	Low risk	Low risk	Low risk	Low risk	Low risk	Low risk	Low risk	Low risk
McKimm, [36] 2018	No information	Serious risk	Low risk	Low risk	No information	Moderate risk	Moderate risk	Serious risk
Palamara, [37] 2015	Serious risk	Moderate risk	Low risk	Low risk	No information	Moderate risk	Moderate risk	Serious risk
Palamara, [38] 2018	Moderate risk	Moderate risk	Moderate risk	Moderate risk	No information	Moderate risk	Moderate risk	Serious risk
Palamara, [39] 2021	Moderate risk	Moderate risk	Moderate risk	Moderate risk	No information	Low risk	Low risk	Moderate risk
Solms, [41] 2021	Moderate risk	Moderate risk	Low risk	Low risk	No information	Low risk	Low risk	Moderate risk
Song, [42] 2020	Moderate risk	Moderate risk	Moderate risk	Moderate risk	Moderate risk	Moderate risk	Moderate risk	Serious risk

### Definition of coaching and qualifications of coaches

Each of the included studies fell within the ICF definition of coaching, focusing on participant-centered goals, a collaborative coach-participant relationship, strengths-development, and maximizing participants’ potential or performance (Table 2). Ten studies specifically indicated that the coaches involved were accredited by a coaching organization or had professional coaching certifications. The other 4 studies indicated that coaches were trained but provided limited certification details. Details about coaches’ certification for individual studies are included in Table 1.

### Coaching intervention and control description

The median number of coaching sessions involved was 5.5 (IQR = 2.75) (Table 5). Two studies did not report the number of sessions and were excluded from this calculation. Coaching sessions ranged in duration from 30 minutes to a full day. Six studies included a control group, where participants received no intervention at all or were offered coaching at the end of the study (Table 5). One study included a control group that received wellness resources via email during the study period. The other 7 studies did not include a control group (Table 5). Coaching was delivered at the group level in four studies [30, 32, 33, 42], and at the individual level by the remaining 10 studies.

**Table 5:** Effectiveness of coaching on physician well-being, distress and burnout.

First author, year	Study design, sample size	Coaching intervention (number, duration, frequency of sessions) vs. control	Outcome(s), measure, timing	Results
de Lasson, [30] 2016	Qualitative study, 45	8, three whole-day sessions and five 2 h sessions, NR Control: NA	Adoption to medical culture, career planning, work/life balance; thematic analysis; halfway point (2 months) and end of intervention (4 months)	“Participants typically stated that they had gained a new awareness of their patterns of thinking, feelings and reactions and found new ways of taking control of their professional lives. Participants were more at ease with themselves.”
Dyrbye, [19] 2019	Randomized controlled trial, 88	5, initial: 1 hour; remainder: 30 minutes, every 2–3 weeks over 5 months Control: No coaching intervention (but given access to coaches after 5 months).	Burnout: Maslach Burnout Inventory; Baseline and end of study (5 months)	Prevalence of symptoms decreased by 17.1% in the intervention and increased by 4.9% in the control group; absolute change -22.0 (95% CI, -25.2 to -18.7), p<0.001
			Quality of life; Single-item linear analog scale; Baseline and end of study (5 months)	Improved in intervention group: absolute change mean score (SD) = 1.2 (2.5) vs control group = 0.1 (1.7); absolute change, intervention vs. control = 1.1 (95% CI, 0.04 to 1.21), p = 0.005
			Resilience; Connor-Davidson Resilience Scale; Baseline and end of study (5 months)	Improved in intervention group: absolute change mean score (SD) = 1.3 (5.2) vs. control group = 0.6(4.0); absolute change intervention vs. control group = 0.7 (95% CI, 0.0 to 3.0), p = 0.04
			Job satisfaction; Global job satisfaction subscale of the Physician Job Satisfaction Scale; Baseline and end of study (5 months)	No difference (p = 0.79)
			Work engagement; Utrecht Work Engagement Scale; Baseline and end of study (5 months)	No difference (Vigor: p = 0.16; Dedication: p = 0.73; Absorption: p = 0.77)
			Empowerment at work; Empowerment at Work Scale; Baseline and end of study (5 months)	No difference (p = 0.95)
Dyrbye, 2022 [31]	Randomized controlled trial, 80	6, initial: 1 hour; remainder: 30 minutes, every 2–3 weeks over 5 months Control: No coaching intervention (but given access to coaches after 6 months).	Burnout: Maslach Burnout Inventory (MBI); Baseline and end of study (6 months)	Decreased by 2.5% in intervention group; increased by 2.5% control group (delta -5.0%, 95% CI -8.6%, -1.4%, p = 0.007).
			Quality of life; Single-item linear analog scale; Baseline and end of study (6 months)	No statistically significant difference.
			Resilience; Connor-Davidson Resilience Scale; Baseline and end of study (6 months)	Increased by 1.9 points in intervention group; decreased by 0.2 points in control group (delta 2.2 points, 95% CI0.1, 4.3, p = 0.04).
			Depersonalization; Subscale of MBI (6 months)	Greater reduction in intervention group vs. control group: mean (SD) = -1.3 (3.1) points vs. 0.4 (3.6) points, delta -1.7 points, 95% CI -3.2, -0.2; p = 0.03). Rates of high depersonalization decreased by 3.8% in intervention group; increased by 2.5% in control group (delta -6.3%, 95% CI -9.5%, -3.1%; p <0.001).
			Emotional exhaustion; Subscale of MBI (6 months)	Rates of high emotional exhaustion decreased by 5.4% in intervention group and by 2.5% in control group (delta -2.9%, 95% CI -6.4%, 0.7%; p = 0.11).
Fainstad, 2022 [32]	Randomized controlled trial, 101	Intervention: Participants could participate in any or all of the following over a 6-month period: (1) two 1-hour group coaching calls per week; unlimited anonymous written coaching via online forum; unlimited access to weekly self-study modules Control: No coaching intervention (but offered coaching program after 6 months).	Burnout: Maslach Burnout Inventory subscales of Emotional Exhaustion (EE), Depersonalization (DP), Professional Accomplishment (PA); Baseline and end of study (6 months)	EE lower in intervention group, increased in control group: mean (SE) score = -3.26 (1.25) vs. 1.07 (1.12), p = 0.01 DP: No statistically significant effect for intervention or control group (−1.06 [0.64] vs −0.03 [0.58]; P = .23) PA: No statistically significant effect for intervention or control group (1.16[0.83] vs 0.25 [0.75]; P = .41)
			Imposter syndrome: Young Imposter Syndrome Scale; Baseline and end of study (6 months)	Reduced in intervention group; increased in control group: mean (SE) score = −1.16(0.31) vs 0.11 (0.27), p = 0.003
			Self-compassion: Neff’s Self-Compassion Scale—Short Form; Baseline and end of study (6 months)	Improved in intervention group: mean (SE) score = 5.5(0.89) vs. -1.32 (0.80), p<0.001
			Moral Injury: Moral Injury Symptom Scale—Healthcare professionals; Baseline and end of study (6 months)	No statistically significant effect for intervention or control group: mean(SE) score = -5.39 (1.62) vs. -183 (1.47), p = 0.10
Gardiner, [33] 2013	Non-randomized experimental study, 69	8 coaching workshops and 6 weeks of email coaching, 9-hours total, Over 3 year period Control: No coaching intervention	Distress; 10-item unnamed scale; before and 3 to 42 months after the intervention	Lower in intervention group: Mean score = 24.50 (95% CI, 21.71–27.29) vs. control group: mean score = 28.63 (95% CI, 27.08–30.17)
			Intention to leave practice; 7-item unnamed scale; before and 3 to 42 months after the intervention	Before coaching, 81% of participants in the intervention group had considered leaving general practice; decreased to 40% after coaching (c2(2) = 16.31, P < .001).
			Retention rate; calculated by comparing coaching participants with the total remaining population of rural physicians (n = 312); 2 time points, 3 years apart.	“Over a 3-year period, 94% of the coaching group remained in general practice compared with 80% of the control group (c2(1) = 4.89, P = 0.027).”
Kakarala, [34] 2018	Pre-post study design, 12	NR, 1 year program, NR Control: NA	Emotional exhaustion; subscale of Maslach Burnout Inventory; Baseline and 1-year.	Emotional exhaustion was high or medium for 60% of participants at baseline, and 56% at 1-year.
McGonagle, [35] 2020	Randomized controlled trial, 58	6, First session: 60min; remainder: 30 min, approximately every 2 weeks Control: No coaching intervention	Burnout; Maslach Burnout Inventory; Pre- and post-intervention and 3- and 6-months post-intervention.	Intervention group vs. control group (p = 0.003): F = 9.82** Pre: M(SD) = 2.32 (0.68) vs. 2.37 (0.71) Post: M(SD) = 1.97 (0.72) vs. 2.45 (0.72)
			Work stress; 15-item Stress in General Scale; Pre- and post-intervention and 3- and 6-months post-intervention.	Intervention group vs. control group (p = 0.077): Pre: M(SD) = 2.04 (0.77) vs. 2.13 (0.45) Post: M(SD) = 1.72 (0.76) vs. 2.09 (0.57)
			Turnover intentions; 3-item Turnover Intentions scale; Pre- and post-intervention and 3- and 6-months post-intervention.	Intervention group vs. control group (p = 0.062): Pre: M(SD) = 2.05 (0.91) vs. 1.75 (0.67) Post: M(SD) = 1.82 (0.85) vs. 1.76 (0.76)
			Engagement; 17-item Engagement Scale; Pre- and post-intervention and 3- and 6-months post-intervention. Psychological capital; 24-item Psychological Capital Questionnaire; Pre- and post-intervention and 3- and 6-months post-intervention.	Intervention group vs. control group (p = 0.023): F = 5.49** Pre: M(SD) = 5.73 (0.78) vs. 5.87 (0.88) Post: M(SD) = 6.06 (0.68) vs. 5.92 (0.68) Intervention group vs. control group (p = 0.002): f = 10.39** Pre: M(SD) = 4.08 (0.69) vs. 4.23 (0.68) Post: M(SD) = 4.63 (0.68) vs. 4.39 (0.74)
			Compassion; 5-item Santa Clara	Intervention group vs. control group (p = 0.784):
			Brief Compassion Scale; Pre- and post-intervention and 3- and 6-months post-intervention.	Pre: M(SD) = 5.47 (0.93) vs. 5.48 (0.99) Post: M(SD) = 5.63 (0.83) vs. 5.58(1.01)
			Job self-efficacy; 7-item Job Self-Efficacy Scale; Pre- and post-intervention and 3- and 6-months post-intervention.	Intervention group vs. control group (p = 0.62): Pre: M(SD) = 3.78 (0.66) vs. 3.46 (0.72) Post: M(SD) = 4.01 (0.60) vs. 3.61 (0.70)
			Job satisfaction; three items from Cammann, Fichman, Jenkins, and Klesh Scale; Pre- and post-intervention and 3- and 6-months post-intervention.	Intervention group vs. control group (p = 0.021): F = 5.74** Pre: M(SD) = 3.59 (0.95) vs. 4.11 (0.54) Post: M(SD) = 3.91 (0.80) vs. 4.04 (0.53)
McKimm, [36] 2018	Mixed-methods study (pre/post design + qualitative interviews), 52	4, NR, a period of up to 18 months. Control: NA	Likelihood of leaving profession; unnamed 10-point scale; baseline and immediately after intervention	“Pre-coaching, 75% of respondents said they were likely (score of 7+) to leave general practice; this fell to 21% (nine GPs) of post-coaching questionnaire respondents. Of the latter group, half of them were aged between 50 and –60 and 4 planned to continue working as a doctor in different roles.”
			Performance under pressure; Human Function Curve; first and last coaching sessions.	“At the start of coaching, 4 GPs rated themselves at breakdown point, 27 at the point of exhaustion and a further 4 considered themselves fatigued. GPs’ self-rated performance was considerably improved at the end of coaching. The final ratings of all except 4 GPs moved from ‘distress’, ‘boredom’ and excess pressure nearer to the ‘safe zone’, with 27 moving to the ‘safe zone’. Only one GP moved further up the ‘pressure scale’; however, they explained that coaching had revealed they were in denial about the pressure they were working under at the start of coaching.”
Palamara, [37] 2015	Pre-post study design, 72	Up to 4, 40 minutes, every 3 months Control: NA	Personal accomplishment; subscale of Maslach Burnout Inventory; Baseline, 3-months, 12-months (post)	High personal accomplishment: pre-coaching 49/59 participants; post-coaching: 48/59 participants
			Emotional exhaustion; subscale of Maslach Burnout Inventory; Baseline, 3-months, 12-months (post)	High emotional exhaustion: pre-coaching: 44/59 participants; post-coaching: 33/59 participants
Palamara, [38] 2018	Pre-post study design, 104	3 or more per year, NR, every 3 months Control: NA	Coping skills; unnamed survey; 3 years	70% of those who participated fully in the coaching program indicated improved coping skills
			Perception of professional relationships; unnamed survey; 3 years	70% of those who participated fully in the coaching program indicated improved relationships
			Emotional exhaustion; subscale of Maslach Burnout Inventory; 3 years	62.8% of participants who reported excellent opportunities for reflection with their coach also reported lower levels of emotional exhaustion
Palamara, [37] 2021	Pre-post study design, 129	2–3 per year, 45–60 minutes, every 3 months Control: NA	Burnout; Depersonalization and Emotional exhaustion subscales of Maslach Burnout Inventory; baseline and 8 months	Participants with higher burnout (measured by depersonalization and emotional exhaustion scores) at baseline more likely to improve and experience lower burnout at 8 months (-0.393, p<0.001; -0.476, p<0.001).
			Emotional exhaustion; subscale of Maslach Burnout Inventory; baseline and 8 months	Black/Asian/Hispanic participants more likely to experience increase compared to non-Hispanic white participants (2.608, 0.46). Decrease for non-Hispanic white participants (mean difference = -1.86, p = 0.02)
			Well-being; PERMA Well-being Scale; baseline and 8 months	Well-being changed from baseline to follow-up in all participants; females showed a decline while males showed an increase (−1.41 vs. 0.83, p = 0.04). Less improvement observed if participant had higher well-being at baseline (-0.407, p<0.001).
Palamara, [40] 2022	Randomized controlled trial, 150	Intervention: Minimum 3 coaching sessions, 45–60 minutes, 9-month period Control: three emails over study period containing well-being resources	Professional fulfillment; Professional fulfillment index (PFI) subscale; baseline and post-intervention (9 months)	Increase: mean (SD) pre 2.33(0.67) vs post 2.52(0.77) p = 0.021; Cohen’s d = 0.26
			Burnout (work exhaustion + interpersonal disengagement); PFI subscale; baseline and post-intervention (9 months)	Decrease in burnout: mean (SD) pre 1.39(0.68) vs post 1.19(0.58) p = 0.026; Cohen’s d = -0.26], Decrease in work exhaustion: mean (SD) pre 1.72(0.78) vs post 1.48(0.67) p = 0.017; Cohen’s d = -0.27 Decrease in interpersonal disengagement: mean (SD) pre 1.17(0.71) vs post 1.00(0.62) p = 0.071; Cohen’s d = -0.21
			Self-evaluation; unnamed 5-point Likert scale; baseline and post-intervention (9 months)	Increase: mean (SD) pre 1.42(0.71) vs post 1.75(0.82) p = 0.0003; Cohen’s d = 0.35
			Positive emotions, engagement, relationship, meaning and accomplishment (PERMA); PERMA scale; baseline and post-intervention (9 months)	Increase: mean (SD) pre 55.47(7.60) vs post 58.05(7.90) p = 0.002; Cohen’s d = 0.37
			Intolerance of Uncertainty (IUS); IUS scale; baseline and post-intervention (9 months)	Improvement (reduction in IUS score) in intervention group (53.0% to 40.8%), but not statistically significant (p = 0.07)
			Resilience: Hardiness Resilience Score scale; baseline and post-intervention (9 months)	No significant change
Solms, [41] 2021	Non-randomized experimental study, 114	6, 60–90 minutes, varied (time in between sessions determined by participants) Control: No coaching intervention	Emotional exhaustion; subscale of Maslach Burnout Inventory; baseline and 10 months	Pre: mean(SD) = 2.75 (1.08) Post: mean(SD) = 2.25 (0.79), p = 0.000
			Cynicism; subscale of Maslach Burnout Inventory; baseline and 10 months	Pre: mean(SD) = 2.11(1.08) Post: mean(SD) = 1.90 (SD 0.75), p = 0.151
			Work engagement; Utrecht Work Engagement Scale; baseline and 10 months	Pre: mean(SD) = 5.08(0.78) Post: mean(SD) = 5.28 (0.59), p = 0.033
			Psychological capital; PsyCap questionnaire; baseline and 10 months	Pre: mean (SD) = 4.83(0.69) Post: mean (SD) = 5.16(0.65), p = 0.000
			Self-compassion; Self-Compassion Scale; baseline and 10 months	Pre: mean (SD) = 3.07(0.60) Post: mean (SD) = 3.27(0.52), p = 0.009
			Psychological flexibility; Work Acceptance and Action Questionnaire; baseline and 10 months	Pre: mean (SD) = 3.43(0.63) Post: mean (SD) = 3.47(0.65), p = 0.6
Song, [42] 2020	Mixed-methods study (pre/post design + qualitative interviews), 25	1 group workshop (2 hours) + 8 individual sessions (1 hour), spaced throughout academic year Control: NA	Burnout; Abbreviated Maslach Burnout Inventory; first and last coaching sessions	No statistically significant changes in burnout scores or the proportion of participants at risk of burnout before (60%) and after (52%) coaching (p = 0.78).
			Resilience; Brief Resilience Scale; first and last coaching sessions	Mean resilience score improved: mean (SD) = 3.8 (0.8) to 4.2 (0.7) (p = 0.002). Improvement was observed primarily among men (mean [SD], 4.1 [0.7] vs. 4.5 [0.5], p = 0.007) rather than women (mean [SD], 3.4 [1.0] vs 3.6 [0.8], p = 0.16).
			Positive and negative feelings; Scale of Positive and Negative Experience; first and last coaching sessions	No statistically significant improvement (mean [SD] = 6.7 [8.2] vs 8.4 [8.3], p = 0.14)
			Perceptions of coaching experience, burnout and wellness; grounded theory analysis; end of intervention	Most participants reported that coaching provided useful skills and was helpful for improve well-being, but felt it should be offered for a longer duration.

### Effectiveness of coaching for improving well-being, distress and burnout

Of the 13 included studies with a quantitative component, 7 reported a decrease in emotional exhaustion (n participants = 612 [58%]) and 5 reported a decrease in overall burnout (n participants = 505 [48%]) (Table 5). Four of the 5 studies that found a decrease in burnout were RCTs (Table 5). McGonagle et al. [35] reported a mean burnout score of 1.97 (baseline: 2.32) for the intervention group compared to 2.45 (baseline: 2.37) for the control group (p = 0.003). Dyrbye et al. [19] reported that the prevalence of symptoms decreased by 17.1% in the intervention group and increased by 4.9% in the control group (absolute change = -22.0 [95% CI, -25.2 to -18.7], p<0.001). In their 2022 study, Dyrbye et al. [31] reported a 2.5% decrease in burnout in the intervention group, whereas burnout increased in the control group (p = 0.007). Palamara et al. [40] reported a decrease in burnout score from 1.39 to 1.19 for participants who received coaching (p = 0.026). The remaining studies either did not assess these outcomes or reported no difference (Table 5).

Increased resilience of coaching participants was observed by three studies (n participants = 193 [18%]). Each of the outcomes—psychological capital (n participants = 172 [16%]), work engagement (n participants = 172 [16%]), emotional well-being (n participants = 279 [26%]) and job retention (n participants = 190 [18%])—were reported to improve after participation in the coaching intervention by 2 studies (Table 5). Improvements in compassion (n participants = 114 [11%]), coping skills (n participants = 104 [10%]), job satisfaction (n participants = 58 [6%]), perception of professional relationships (n participants = 104 [10%]), performance under pressure (n participants = 52 [5%]), quality of life (n participants = 88 [8%]), self-compassion (n participants = 101 [10%]), professional fulfillment (n participants = 150 [14%]), self-evaluation (n = 150 [14%]) were each observed by 1 study (Table 5). Reduction in imposter syndrome (n participants = 101 [10%]), work exhaustion (n participants = 150 [14%]), interpersonal disengagement (n participants = 150 [14%]), depersonalization (n participants = 80 [8%]), intolerance of uncertainty (n participants = 150 [14%]) were each observed by one study. One study also reported that positive changes in well-being after coaching were experienced only by Caucasian and male participants [39].

In the sole included qualitative study by de Lasson et al., [30] participants reported gaining “a new awareness of their patterns of thinking, feelings and reactions” and being “more at ease with themselves”. Participants also reported finding new ways to take control of their professional lives (Table 5).

## Discussion

### Summary of main findings

This systematic review identified 14 studies assessing the effect of coaching on physician wellness and burnout. Five of these studies were RCTs at relatively low risk of bias, while the included non-randomized interventional studies were mostly at moderate or serious risk of bias. Across all studies, coaching was observed to improve several outcomes related to wellness, including work/life balance, quality of life, resilience, job satisfaction, work engagement, empowerment at work, and psychological capital. Coaching was also generally observed to decrease emotional exhaustion, distress, and burnout.

The definition of coaching used in included studies was relatively homogeneous, as could be expected based on our inclusion criteria which rigorously adhered to the International Coaching Federation definition of coaching [20]. Although studies viewed coaching similarly, their implementation of coaching varied. For example, some studies involved only 3 to 4 sessions while others involved 6 or more. The duration of the sessions also differed between studies as well as the time period over which coaching was offered and whether coaching was delivered at the group or individual level. Despite this variation, coaching still appeared to positively impact participant wellness regardless of its mode of delivery. It may be worthwhile to explore the minimally effective “dose” of coaching in future research to maximize benefits for physician wellness while working within resource constraints and scheduling challenges. In addition, the optimal level of coach certification for maximizing physician wellness should be explored as it also varied between the studies included here. Currently, coaching is an unregulated profession, albeit there are national and international certification bodies. Minimum standards of coach competency and conduct may be in participants’ best interest moving forward if there is a relationship between coach certification and participant outcomes.

In order to effectively make comparisons, standardized reporting and outcome measures are also needed. The included studies assessed a wide range of outcome measures and designs. Baseline and post-intervention proportions of the outcome measures were also not regularly reported. The heterogeneity in outcome measures among the included studies may reflect the lack of consensus in the broader literature regarding the best way to conceptualize and assess physician wellness [43]. Given the multifaceted nature of physician wellness, there is a need for “a shared, holistic definition… that explicitly includes integrated well-being constructs (e.g., purpose, thriving, vigor, work-life balance) along with mental, social and physical constructs.” [43] A comprehensive and shared definition will improve the quality of research on physician wellness, including studies of intervention effectiveness, and increase comparability of findings.

Another consideration for reporting is to disaggregate results by relevant participant characteristics, such as sex, gender, and ethnicity. One of the included studies observed differential effectiveness of coaching by sex and ethnicity of participants, while other studies did not report results across these variables. Recent studies document that the prevalence, presentation, and factors contributing to burnout can vary by race/ethnicity, gender, and sexual orientation [44–48]. Consequently, these and other equity-related characteristics may be important to consider when evaluating the outcomes of coaching. Interestingly, two of the included RCTs evaluated the effectiveness of coaching for female participants only, and found significant benefits [44, 45]. Qualitative research may be useful to explore the mechanisms of change among coaching participants and generate hypotheses regarding its relative effectiveness within and across groups of physicians.

### Strengths and limitations

This review identifies five RCTs at relatively low risk of bias that show evidence supporting the effectiveness of coaching for physicians. There are some limitations based on inconsistent and incomplete reporting were common across the included studies. In addition, most studies were not RCTs, and these were all at moderate to serious risk of bias. Nevertheless, this review addresses an emerging and promising intervention for physician wellness at a time when levels of distress and burnout are on the rise. It also identifies key gaps for future research on coaching to address.

### Implications for researchers, clinicians and policymakers

Based on the available evidence, coaching may be a promising intervention for improving physician wellness but requires further study to precisely determine its optimal delivery and effectiveness. Additional RCTs with standardized reporting and outcome measures, sub-group analyses, and qualitative exploratory work are needed. At the same time, coaching may be an appealing intervention to clinicians given its flexibility and strengths-based approach. Should future research conclusively support its effectiveness for improving physician wellness, healthcare organizations may wish to facilitate coaching as part of their wellness programs for physicians.

## Conclusions

Evidence from available RCTs suggests coaching for physicians can improve well-being and reduce distress/burnout. Non-randomized interventional studies have similar findings but face many limitations. Consistent reporting and standardized outcome measures are needed.

## Supporting information

S1 Appendix Search strategies. (DOCX)

S1 Checklist PRISMA checklist. (DOCX)
