# Supplementary material for: Impact of coaching on physician wellness: A systematic review
Source: PLoS One. 2023 Feb 7;18(2):e0281406. doi: 10.1371/journal.pone.0281406 (PMC9904500; doi:10.1371/journal.pone.0281406)
Supplement: S1 Appendix — (DOCX) [file pone.0281406.s001.docx]

S1 Appendix. Search Strategies

| **Medline (Ovid) Search Strategy (1946 to December 21, 2021)** | | |
| --- | --- | --- |
| **#** | **Searches** | **Results** |
| 1 | stress, psychological/ | 129047 |
| 2 | burnout, psychological/ | 1108 |
| 3 | burnout, professional/ | 14590 |
| 4 | psychological distress/ | 2761 |
| 5 | occupational stress/ | 2852 |
| 6 | compassion fatigue/ | 693 |
| 7 | anxiety/ | 94194 |
| 8 | (stress* or distress* or burnout* or burn*-out* or anxi*).ti,ab,kf. | 1275448 |
| 9 | ((mental* or emotion* or psych*) adj3 (strain* or exhaust* or tense* or tension* or pressur* or adjust* or adapt*)).ti,ab,kf. | 26719 |
| 10 | ((compassion* or mental*) adj2 fatigue*).ti,ab,kf. | 2755 |
| 11 | ((vicarious* or second*) adj2 trauma*).ti,ab,kf. | 3684 |
| 12 | adaptation, psychological/ | 100209 |
| 13 | mental health/ | 49666 |
| 14 | (well-being or wellbeing or wellness or cope? or coping or resilien*).ti,ab,kf. | 252170 |
| 15 | (mental* adj2 (health* or hygiene)).ti,ab,kf. | 190503 |
| 16 | or/1-15 | 1680078 |
| 17 | exp physicians/ | 160303 |
| 18 | exp education, medical, graduate/ | 76582 |
| 19 | education, medical, continuing/ | 25359 |
| 20 | (doctor? or physician* or surgeon* or general practitioner* or residenc* or resident? or intern? or internship* or fellow*).ti,ab,kf. | 1029634 |
| 21 | ((postgraduate or graduate or continuing) adj2 (medicine or medical) adj2 (educat* or instruct* or program* or teach* or train*)).ti,ab,kf. | 16644 |
| 22 | or/17-21 | 1127664 |
| 23 | mentoring/ | 2957 |
| 24 | coach*.ti,ab,kf. | 17688 |
| 25 | or/23-24 | 19500 |
| 26 | 16 and 22 and 25 | 416 |

| **Embase (Ovid) Search Strategy (1947 to December 21, 2021)** | | |
| --- | --- | --- |
| **#** | **Searches** | **Results** |
| 1 | burnout/ | 21359 |
| 2 | professional burnout/ | 1689 |
| 3 | mental stress/ | 93598 |
| 4 | emotional stress/ | 24895 |
| 5 | exp job stress/ | 12754 |
| 6 | distress syndrome/ | 52307 |
| 7 | anxiety/ | 247037 |
| 8 | (stress* or distress* or burnout* or burn*-out* or anxi*).ti,ab,kf. | 1713429 |
| 9 | ((mental* or emotion* or psych*) adj3 (strain* or exhaust* or tense* or tension* or pressur* or adjust* or adapt*)).ti,ab,kf. | 35406 |
| 10 | ((compassion* or mental*) adj2 fatigue*).ti,ab,kf. | 3687 |
| 11 | ((vicarious* or second*) adj2 trauma*).ti,ab,kf. | 4995 |
| 12 | psychological adjustment/ | 1571 |
| 13 | mental health/ | 167962 |
| 14 | wellbeing/ | 74281 |
| 15 | emotional well-being/ | 1135 |
| 16 | psychological well-being/ | 23702 |
| 17 | professional well-being/ | 130 |
| 18 | (well-being or wellbeing or wellness or cope? or coping or resilien*).ti,ab,kf. | 317427 |
| 19 | (mental* adj2 (health* or hygiene)).ti,ab,kf. | 233117 |
| 20 | or/1-19 | 2253174 |
| 21 | exp physician/ | 886901 |
| 22 | resident/ | 55263 |
| 23 | medical education/ | 248465 |
| 24 | residency education/ | 30308 |
| 25 | surgical training/ | 23552 |
| 26 | (doctor? or physician* or surgeon* or general practitioner* or residenc* or resident? or intern? or internship* or fellow*).ti,ab,kf. | 1494522 |
| 27 | ((postgraduate or graduate or continuing) adj2 (medicine or medical) adj2 (educat* or instruct* or program* or teach* or train*)).ti,ab,kf. | 20815 |
| 28 | or/21-27 | 2052553 |
| 29 | mentor/ | 7786 |
| 30 | coach*.ti,ab,kf. | 22583 |
| 31 | or/29-30 | 30092 |
| 32 | 20 and 28 and 31 | 1241 |

| **APA PsycINFO (Ovid) Search Strategy (1806 to December Week 3 2021)** | | |
| --- | --- | --- |
| **#** | **Searches** | **Results** |
| 1 | exp occupational stress/ | 23756 |
| 2 | Stress/ | 68409 |
| 3 | Academic Stress/ | 637 |
| 4 | chronic stress/ | 2956 |
| 5 | Posttraumatic Stress/ | 1258 |
| 6 | Psychological Stress/ | 9260 |
| 7 | Distress/ | 25343 |
| 8 | Anxiety/ | 68443 |
| 9 | (stress* or distress* or burnout* or burn*-out* or anxi*).tw. | 533135 |
| 10 | ((mental* or emotion* or psych*) adj3 (strain* or exhaust* or tense* or tension* or pressur* or adjust* or adapt*)).tw. | 37994 |
| 11 | ((compassion* or mental*) adj2 fatigue*).tw. | 2318 |
| 12 | ((vicarious* or second*) adj2 trauma*).tw. | 2722 |
| 13 | Emotional Adjustment/ | 16774 |
| 14 | Occupational Adjustment/ | 2045 |
| 15 | "Resilience (Psychological)"/ | 17223 |
| 16 | Psychological Endurance/ | 684 |
| 17 | Mental Health/ | 76009 |
| 18 | exp Well Being/ | 51445 |
| 19 | (well-being or wellbeing or wellness or cope? or coping or resilien*).tw. | 242490 |
| 20 | (mental* adj2 (health* or hygiene)).tw. | 218575 |
| 21 | or/1-20 | 871115 |
| 22 | exp Physicians/ | 46103 |
| 23 | exp Medical Education/ | 25383 |
| 24 | (doctor? or physician* or surgeon* or general practitioner* or residenc* or resident? or intern? or internship* or fellow*).tw. | 179924 |
| 25 | ((postgraduate or graduate or continuing) adj2 (medicine or medical) adj2 (educat* or instruct* or program* or teach* or train*)).tw. | 2214 |
| 26 | or/22-25 | 206205 |
| 27 | exp Coaching/ | 9144 |
| 28 | Coaching Psychology/ | 547 |
| 29 | coach*.tw. | 20664 |
| 30 | or/27-29 | 20965 |
| 31 | 21 and 26 and 30 | 198 |

| **ERIC (Ovid) Search Strategy (1965 to May 2021)** | | |
| --- | --- | --- |
| # | **Searches** | **Results** |
| 1 | Burnout/ | 1334 |
| 2 | stress variables/ | 13063 |
| 3 | Anxiety/ | 13343 |
| 4 | Posttraumatic Stress Disorder/ | 1774 |
| 5 | (stress* or distress* or burnout* or burn*-out* or anxi*).tw. | 68105 |
| 6 | ((mental* or emotion* or psych*) adj3 (strain* or exhaust* or tense* or tension* or pressur* or adjust* or adapt*)).tw. | 6311 |
| 7 | ((compassion* or mental*) adj2 fatigue*).tw. | 91 |
| 8 | ((vicarious* or second*) adj2 trauma*).tw. | 141 |
| 9 | coping/ | 12381 |
| 10 | emotional adjustment/ | 3460 |
| 11 | vocational adjustment/ | 1910 |
| 12 | "resilience (psychology)"/ | 3562 |
| 13 | mental health/ | 13124 |
| 14 | well being/ | 11163 |
| 15 | wellness/ | 1418 |
| 16 | (well-being or wellbeing or wellness or cope? or coping or resilien*).tw. | 45174 |
| 17 | (mental* adj2 (health* or hygiene)).tw. | 22133 |
| 18 | or/1-17 | 122291 |
| 19 | exp Physicians/ | 4391 |
| 20 | medical education/ | 9786 |
| 21 | Graduate Medical Education/ | 1305 |
| 22 | (doctor? or physician* or surgeon* or general practitioner* or residenc* or resident? or intern? or internship* or fellow*).tw. | 43532 |
| 23 | ((postgraduate or graduate or continuing) adj2 (medicine or medical) adj2 (educat* or instruct* or program* or teach* or train*)).tw. | 1877 |
| 24 | or/19-23 | 50318 |
| 25 | exp "coaching (performance)"/ | 4043 |
| 26 | coach*.tw. | 12046 |
| 27 | or/25-26 | 12046 |
| 28 | 18 and 24 and 27 | 26 |

| **Scopus Search Strategy** | | |
| --- | --- | --- |
| **#** | **Searches** | **Results** |
| 1 | TITLE-ABS-KEY ( stress* OR distress* OR burnout* OR burn*-out* OR anxi* ) | 3523416 |
| 2 | TITLE-ABS-KEY ( ( mental* OR emotion* OR psych* ) W/3 ( strain* OR exhaust* OR tense* OR tension* OR pressur* OR adjust* OR adapt* ) ) | 137998 |
| 3 | TITLE-ABS-KEY ( ( compassion* OR mental* ) W/2 fatigue* ) | 6335 |
| 4 | TITLE-ABS-KEY ( ( vicarious* OR second* ) W/2 trauma* ) | 7859 |
| 5 | TITLE-ABS-KEY ( well-being OR wellbeing OR wellness OR cope? OR coping OR resilien* ) | 584128 |
| 6 | TITLE-ABS-KEY ( mental* W/2 ( health* OR hygiene ) ) | 388927 |
| 7 | TITLE-ABS-KEY ( doctor? OR physician* OR surgeon* OR "general practitioner*" OR residenc* OR resident? OR intern? OR internship* OR fellow* ) | 1724438 |
| 8 | TITLE-ABS-KEY ( ( postgraduate OR graduate OR continuing ) W/2 ( medicine OR medical ) W/2 ( educat* OR instruct* OR program* OR teach* OR train* ) ) | 63114 |
| 9 | TITLE-ABS-KEY ( coach* ) | 45549 |
| 10 | (#1 OR #2 OR #3 OR #4 OR #5 OR #6) AND (#7 OR #8) AND #9 | 546 |
